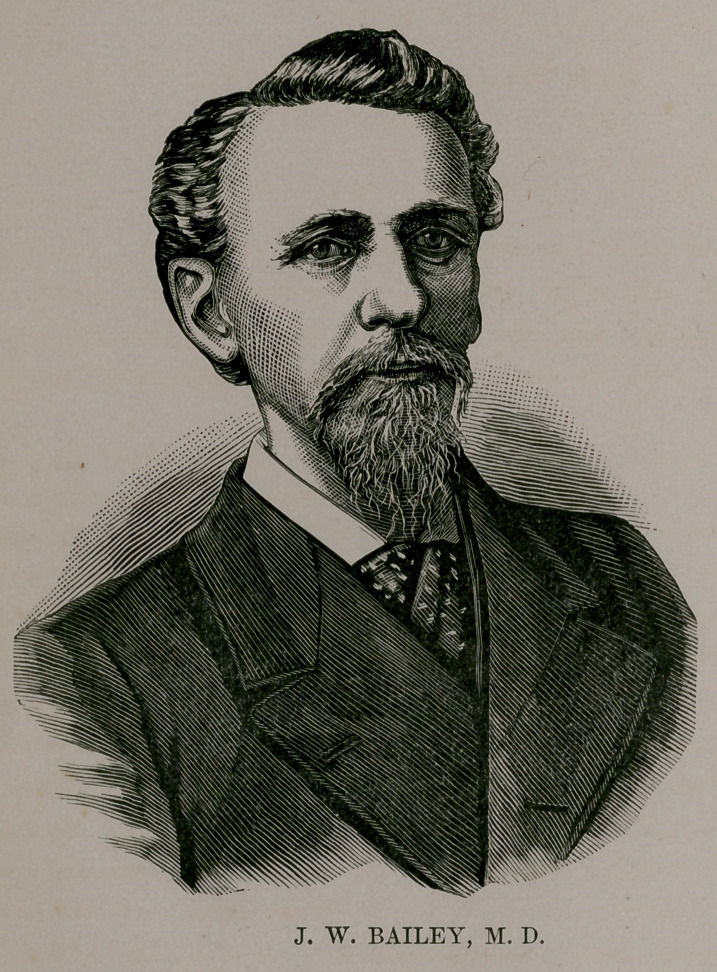# Our Portrait Gallery

**Published:** 1885-05

**Authors:** 


### OUR PORTRAIT GALLERY.

---

### JAMES W. BAILEY, M. D.

James W. Bailey was born in Cleveland county, N. C., in the year 1838. His father, who was a successful farmer, moved to Georgia in 1853, and gave his son the best educational opportunities that were furnished by the common schools of his neighborhood.

At the age of eighteen years, Dr. Bailey commenced the study of medicine under Dr. Ellis, of Forsyth county. This being the profession of his choice, he applied himself closely and assiduously to his text-books, entering the Atlanta Medical College, well advanced, in 1858. During his attendance at the college, he was a close student, and allowed no opportunity to pass unimproved which was likely to aid in his thorough preparation for the practice of medicine. Graduating in 1860, he returned to his home and entered at once upon a large and lucrative practice. This he continued until the beginning of the war between the States, when he promptly responded to the call of his section, and entered the military service of the Southern Confederacy as first sergeant of the Second Georgia Cavalry. Attached to the Western army, he discharged the perilous duties of a gallant soldier for eighteen months, when he was commissioned assistant surgeon of the State troops, and was subsequently made surgeon of a State regiment. He followed the fortunes of General Johnston's army in all of the battles fought in Georgia, rendering valuable service by his ministrations to the sick and wounded soldiers.

After the surrender he returned to Forsyth county and resumed the practice of his profession. He remained there for eight years, enjoying a large practice. At the close of that period, he moved to Gainesville, where he now resides.

Dr. Bailey has taken several special courses in the hospitals of New York, which afforded him excellent opportunities for adding to his extensive acquaintance with the science and practice of medicine and surgery.

He has given much attention to gynecology and diseases of children, attaining distinction in both of these branches of his profession. His learning and experience have rendered his consultation work greater, perhaps, than that of any physician of his section. No higher compliment could be paid him as a learned and successful practitioner. During the summer months, when hundreds of children from other portions of the State are taken to the springs and health resorts of Northeast Georgia, the services of Dr. Bailey are in constant demand.

Being yet in the prime of life, with the prestige of a successful career to encourage him, having amassed a competent fortune by his practice, enjoying the highest confidence and the regard of all good people who know him, he has before him a future full of usefulness and professional fame. Such a man is a fit exemplar for the young physician, and we trust that many who know his history will emulate his life.

## Figures and Tables

**Figure f1:**